# Psychological Distress, Clinical Biomarkers, and Subjective Cognitive Concerns: Implications for Dementia Risk in African Adults

**DOI:** 10.1002/alz70857_102753

**Published:** 2025-12-25

**Authors:** Catherine Bikeri Onyancha, Benard O Aliwa, Jasmit Shah, Stanley Onyango, Litha Musili, ALICE MORAA ONDIEKI, Zul Merali, Mansoor Saleh, Karen Blackmon, Chinedu Udeh‐Momoh

**Affiliations:** ^1^ Brain and Mind Institute, Aga Khan University, Nairobi, Kenya; ^2^ Brain and Mind Institute, Aga Khan University, Nairobi, Nairobi, Kenya; ^3^ Aga Khan University, Nairobi, Nairobi, Kenya; ^4^ Brain and Mind Institute, Aga Khan University, Nairobi, NAIROBI, Kenya; ^5^ Geisel School of Medicine at Dartmouth, Hanover, NH, USA; ^6^ Dartmouth Hitchcock Medical Center, Lebanon, NH, USA; ^7^ Global Brain Health Institute, University of California, San Francisco, NC, USA; ^8^ FINGERS Brain Health Institute, Solna, Stockholm, Sweden; ^9^ Wake Forest University, School of Medicine, Winston‐Salem, NC, USA

## Abstract

**Background:**

Psychological distress is a recognized risk factor for dementia, influencing systemic and cellular aging processes. Subjective cognitive concerns (SCCs) are emerging as early neuropsychiatric symptoms associated with dementia risk. Understanding the interplay between psychological distress, SCCs, and clinical health markers in African populations is critical for developing culturally appropriate interventions to mitigate cognitive decline and systemic health deterioration.

**Method:**

Baseline data were analysed from 145 adults (105 healthy controls, 20 newly diagnosed with cancer, and 20 caregivers) aged 35 years and above, participating in the Brain Resilience Kenya study. Psychological distress was measured using the PHQ‐9 and GAD‐7 scales, while SCCs were assessed using a validated self‐report questionnaire. Clinical biomarkers analysed included fasting glucose, C‐reactive protein, lipid profiles, creatinine, liver function, and thyroid function tests. Statistical models evaluated associations between psychological distress, SCCs, and clinical biomarkers, adjusting for age and gender.

**Result:**

Depressive symptoms were significantly associated with the cholesterol/HDL ratio (*p* = 0.004) and non‐HDL cholesterol (*p* = 0.05). SCCs were significantly linked to depressive symptoms (*p* = 0.03), creatinine levels (*p* = 0.05), and AST levels (*p* = 0.04). However, associations between clinical markers and SCCs were not significant after controlling for age and gender. These findings suggest that while psychological distress and SCCs are associated with certain clinical markers, the observed relationships may be influenced by demographic factors.

**Conclusion:**

This study highlights significant associations between psychological distress, SCCs, and clinical biomarkers, underscoring the need to address mental health as part of dementia prevention strategies. The attenuation of associations after adjusting for age and gender suggests that demographic factors may play a key role in these relationships. Future research should explore longitudinal changes in these markers to better understand their contribution to dementia risk and the development of targeted interventions for African populations.

